# Evaluation of the postoperative stability of a counter-clockwise rotation technique for skeletal class II patients by using a novel three- dimensional position-posture method

**DOI:** 10.1038/s41598-019-49335-2

**Published:** 2019-09-13

**Authors:** Zhuqing Wan, Steve Guofang Shen, Haijun Gui, Peng Zhang, Shunyao Shen

**Affiliations:** 1 Department of Oral and Cranio-maxillofacial Surgery, Shanghai Ninth People’s Hospital, College of Stomatology, Shanghai Jiao Tong University School of Medicine Shanghai Key Laboratory of Stomatology & Shanghai Research Institute of Stomatology No. 639 Zhizaoju Road, Shanghai, 200011 China; 2 2nd Dental Center, Shanghai Ninth People’s Hospital, Shanghai Jiao Tong University School of Medicine Shanghai Key Laboratory of Stomatology No. 639 Zhizaoju Road, Shanghai, 200011 China

**Keywords:** Oral diseases, Craniofacial orthodontics, Orthopaedics

## Abstract

The aim of this study is to evaluate the postsurgical stability of skeletal class II patients after performing a counter-clockwise rotational (CCWR) procedure for the maxilla-mandibular complex (MMC) by using a novel Three-dimensional (3D) Position-Posture(P-P) measuring method. Twenty-five patients (5 males and 20 females) were included in this study. The postoperative CT scans of the skull were taken before surgery(T0), 3–7 days (T1), 3 months (T2), and 6 months (T3) after surgery. Specific anatomic landmarks were chosen to determine the position of the segments, while three equally perpendicular planes were created to describe their posture. The results show that the linear relapse of maxillary landmarks during the follow-up were acceptable (≤0.5 mm). The relapse of maxillary pitch plane at 6-months follow-up is 1.52°, which is acceptable. There was a significant pitch plane relapse of the mandibular-body segment with an average of 1.86° between T1 and T2 models, 3.28° between T1 and T3 models. There was no significant difference between roll and yaw planes during the follow-up. We therefore conclude that the P-P method could be used to accurately analyze the postsurgical stability of skeletal class II orthognathic surgery cases. For CCWR procedures, it was also shown that the there is a tendency for recurrence most specially on the body of the mandible.

## Introduction

Skeletal class II malocclusion is a common dentofacial deformity, which is characterized by the retrusive position of the mandible in relation to the maxilla^1^. Orthognathic surgery with counterclockwise rotation (CCWR) of the maxillo-mandibular complex (MMC) can be performed to achieve a more optimal function and esthetically acceptable outcome^2–4^.

However, postsurgical stability after CCWR remains a challenge. Several studies have reported the poor postsurgical stability after CCWR. A coexisting TMJ pathology can jeopardize the stability of the MMC after performing the CCWR. Because that the increased joint loading can cause TMJ-associated symptoms, condylar resorption, malocclusion, and relapse^5–11^. Wolford^12^ *et al*. reported that there is an increased posterior facial height with subsequent stretching of the pterygoid, masseteric, and suprahyoid muscles that could place additional load to the TMJs, with potential adverse results.

Recently, computer-aided design and computer-aided manufacturing technologies have been developed to improve the repeatability, precision and accuracy of orthognathic surgery, giving the potential to realize ideal CCWR produce during the orthognathic surgery. Meanwhile, the rigid internal fixation technology dramatically improve the postoperative stability of orthognathic surgery^13^. There exist a trade-off for surgeons between the uncertain postoperative stability and ideal aesthetic outcomes. An accurate and objective evaluation system for the postoperative skeletal stability after CCWR produce is needed to minimize the potential risk.

To evaluate the stability after CCWR, traditional methods were often utilised using two-dimensional lateral cephalometric radiographs, which has several disadvantages such as lack of perspective, errors in projection and superimposition, variations in magnification, voids of information, and errors in positioning the cone head^14^.

Three-dimensional (3D) computer-aided surgical simulation system has been evaluated for its clinical feasibility and accuracy^15^. The construction of 3D virtual head models enables the morphologic changes of the skeletal segments more visible^16^. The superimposition of 3D head models realize the quantification of segments movements, which could not been performed on 2D radiographs. The purpose of this study is to establish a novel 3D measuring method to analyse the postoperative skeletal stability after performing the counter-clockwise rotation of the MMC in patients with skeletal class II deformity.

## Results

Twenty-five patients (5 males and 20 females, mean age 23.4 ± 4.7 years, range 18 to 29 years) with skeletal class II deformity were included in this retrospective study. They all underwent othognathic surgery thru the CCWR procedure in our department. The linear plane differences in the landmarks represent the changes in position and the differences in the angle planes represent the changes in posture. The differences in the angle between the left and right ramus is shown in Table 1.

**Table 1:** Statistical analysis of the linear and angular differences of the maxillary segments, mandibular body segments and bilateral ramus planes.

	T0	T1	T1-T0	T2	T2-T1	T3	T3-T1
	Mean (SD)	Mean (SD)	p value	Mean (SD)	p value	Mean (SD)	p value
A-CP (mm)	1.46 (2.91)	−1.65 (3.26)	0.000^※^	−1.28 (3.12)	0.321	−1.17 (2.99)	0.308
A-HP (mm)	62.87 (3.98)	59.44 (4.86)	0.000^※^	59.13 (4.72)	0.866	59.33 (4.70)	1.000
A-SP (mm)	0.30 (1.02)	0.19 (1.23)	1.000	0.14 (1.25)	1.000	−0.03 (1.25)	0.071
SPC-CP (mm)	3.63 (2.53)	2.34 (2.29)	0.024^※^	2.36 (2.29)	1.000	2.13 (1.97)	1.000
SPC-HP (mm)	76.45 (3.82)	72.35 (4.53)	0.000^※^	72.51 (4.51)	1.000	72.32 (4.18)	1.000
SPC-SP (mm)	1.00 (0.91)	0.70 (0.98)	1.000	0.58 (0.93)	1.000	0.54 (0.83)	0.578
SP6L-CP (mm)	−21.71 (3.31)	−23.52 (3.66)	0.022^※^	−23.17 (3.84)	0.600	−23.16 (3.49)	1.000
SP6L-HP (mm)	70.11 (4.90)	68.10 (4.55)	0.002^※^	67.94 (4.86)	1.000	67.86 (4.84)	0.785
SP6L-SP (mm)	29.03 (2.01)	28.85 (2.14)	1.000	28.67 (2.12)	0.340	28.70 (2.21)	1.000
SP6R-CP (mm)	−20.28 (4.43)	−22.51 (3.85)	0.001^※^	−22.08 (3.86)	0.184	−22.06 (3.78)	0.524
SP6R-HP (mm)	70.58 (4.57)	68.56 (4.72)	0.000^※^	68.21 (4.86)	0.118	68.27 (4.80)	0.462
SP6R-SP (mm)	−28.74 (1.54)	−28.56 (1.19)	1.000	−28.60 (1.28)	1.000	−28.55 (0.98)	1.000
∠PP-CP(°)	9.88 (6.31)	16.36 (4.94)	0.000^※^	15.93 (5.19)	1.000	14.84 (5.41)	0.085
∠RP-HP(°)	0.58 (1.95)	0.60 (1.86)	1.000	0.39 (1.91)	0.071	0.37 (1.83)	0.668
∠YP-CP(°)	0.82 (1.97)	0.35 (2.19)	1.000	0.27 (2.46)	1.000	0.32 (2.25)	1.000
∠SNA(°)	84.23 (4.13)	81.11 (4.53)	0.000^※^	81.37 (4.52)	1.000	81.43 (4.57)	0.487
B-CP’ (mm)	71.03 (9.79)	73.84 (8.45)	0.005^※^	73.49 (9.35)	1.000	71.84 (10.25)	0.012^※^
B-HP’ (mm)	57.03 (9.05)	60.01 (9.69)	0.024^※^	60.27 (10.30)	1.000	60.69 (10.45)	1.000
B-SP’ (mm)	54.57 (3.21)	54.53 (2.81)	1.000	54.01 (3.01)	0.161	53.77 (3.06)	0.034^※^
IPC-CP’ (mm)	75.76 (8.45)	77.32 (7.54)	0.221	77.16 (8.63)	1.000	75.53 (9.39)	0.047^※^
IPC-HP’ (mm)	50.05 (10.58)	53.22 (11.29)	0.004^※^	53.42 (11.84)	1.000	54.20 (11.73)	0.329
IPC-SP’ (mm)	54.47 (3.60)	54.44 (3.16)	1.000	53.76 (3.36)	0.025^※^	53.63 (3.30)	0.035^※^
IP6L-CP’ (mm)	54.27 (5.76)	56.97 (3.69)	0.039^※^	56.64 (4.85)	1.000	55.61 (5.34)	0.308
IP6L-HP’ (mm)	45.49 (7.03)	48.42 (7.47)	0.003^※^	48.68 (7.91)	1.000	48.91 (8.08)	1.000
IP6L-SP’ (mm)	80.70 (3.08)	81.19 (2.67)	1.000	80.58 (3.00)	0.055	80.32 (2.79)	0.004^※^
IP6R-CP’(mm)	52.09 (11.67)	54.31 (11.35)	0.026^※^	53.77 (12.49)	0.984	52.53 (12.64)	0.034^※^
IP6R-HP’ (mm)	45.77 (6.98)	49.07 (8.00)	0.002^※^	49.00 (8.06)	1.000	49.47 (8.14)	1.000
IP6R-SP’ (mm)	26.97 (4.74)	27.29 (4.36)	1.000	26.67 (4.49)	0.058	26.44 (4.49)	0.021^※^
∠PP’-CP’(°)	31.16 (12.16)	26.43 (12.16)	0.000^※^	28.29 (11.65)	0.013^※^	29.71 (12.31)	0.000^※^
∠RP’-HP’(°)	1.60 (1.34)	1.57 (1.35)	1.000	1.49 (1.15)	1.000	1.78 (0.95)	1.000
∠YP’-CP’(°)	1.91 (1.24)	1.50 (0.66)	1.000	1.76 (1.15)	1.000	1.66 (1.28)	1.000
∠RsPL-RsPR(°)	25.55 (10.66)	25.21 (10.42)	1.000	24.90 (10.56)	1.000	24.26 (10.48)	0.069

All data showed in mean ± SD.

^※^The difference was considered statistically significant if *p* ≤ 0.05.

It can conclude that the maxilla moved backward and upward postoperatively in the analysis of the difference in measurements between T0 and T1, while all measurements in the mandible had shown that it advanced horizontally (*p* ≤ *0.05* statistically significant difference thru the ANOVA test). During the operation, the change in the mean pitch plane with counterclockwise rotation was 6.48° on the maxilla and 4.73° on the mandible.

Postoperative follow-up showed the linear relapse of the maxillary landmarks between T1, T2 and T3 were less than 0.5 mm, (no statistically significant difference *p* ≤ *0.05*, ANOVA test), which would mean a very stable result. Compared with immediate postoperative maxillary pitch plane posture, there was average 6.64% relapse at 3 months follow-up (T2-T1/T1-T0) and 23.46% relapse at 6 months follow-up (T3-T1/T1-T0). There was no significant angle changes in the roll plane and yaw plane between T1, T2, and T3 models, which is therefore acceptable.

Postoperative follow-up showed that the largest positional linear relapse of the mandibular landmarks were less than 2.0 mm. The distance between the B point and mandibular CP’ increased from 71.03 mm to 73.84 mm postop. Then it further decreased to 73.49 mm three months after surgery, continually decreased to 71.84 mm in the subsequent follow-ups. There was a statistically significant pitch plane relapse of the mandible with an average of 1.86° between T1 and T2, 3.28° between T1 and T3. ∠YP-CP’ showed no significant postural angular changes during the 6-months follow-up.

In conclusion, this novel three-dimensional Position-Posture measuring method can be a useful and reliable tool in measuring the linear and angular changes of target segments and in analyzing the three-dimensional recurrence trend after surgery quamtitatively. Based on the results of the Position-Posture measuring method, it can conclude that the MMC had a clockwise relapse tendency after performing the counter-clockwise procedure for skeletal class II patients, most especially on the mandibular-body segment. Meanwhile, there were no significant changes on each segments on the vertical and transverse dimensions.

## Discussion

Orthognathic surgery using the counterclockwise rotation technique, despite its esthetic and functional advantages, was not used to treat maxillofacial deformities during the mid 1990s because of the uncertain postsurgical stability^1–3^. Most of the previous studies that we encountered have used a two dimensional cephalometric evaluation to measure the postsurgical stability of the orthognathic surgery. All the facial bone structures are projected on a single coronal or sagittal plane in frontal or lateral cephalograms. The tissue superimposition and the changes of the patient’s position may lead to errors in the magnification and unreliability of the linear distance between 2-dimensional landmarks when used as a refs^14,17^.

Computer-assisted-surgery, allowing 3D planning and simulation, offers a new option for 3D measurement and analysis. By reconstructing the skull model, the position changes of bony landmarks and posture changes of bone segments become more visible. However, previous three-dimensional CT measurement system only represent the bony landmarks of craniofacial bone on CT images, but fail to analyze the bone relationship in three dimensional space^18,19^. Xia^20^ *et al*. demonstrated a three-dimensional cephalometry protocol. However, the positon and posture of the segment was defined by geometric method, which is different from the real anatomic landmarks. At the mean time, the error would be dispersed to three axes perpendicular to each other and could not reflect real changes enough. Therefore, the novel “position-posture” method was developed to actually address this need.

For any geometric object, four points and three planes are sufficient to define its position and posture in three dimension. The same principle also can be applied to skull bone segments. This study determined four clinically significant anatomical landmarks which could represent the shape and position of the maxillary or mandibular segments and three postural planes to describe the posture and orientation. The linear and angular changes of the postural plane were measured to analyze the positional and postural changes observed every follow-up. The results of this retrospective study show that postoperative skeletal stability of the maxilla after counterclockwise rotation during orthognathic surgery were acceptable. It also showed that the postoperative angular changes in the pitch plane is more prominent than the other two postural planes. The maxilla also had a clockwise rotation tendency. The linear relapse based on the landmarks on the maxilla during the follow-up revealed no statistical significance. The largest linear changes of mandible-body segments is 2.00 mm at B point, and the pitch plane relapse indicating that the mandible-body segments tend to rotate clockwise compared to bilateral ramus segments at 6 months follow-up.

Many factors including articular diseases, age and sex of the patient, a high angle of the mandibular plane, condylar position, neuromuscular adaptation, instability of fixation, and the amount of forward advancement may contribute to the clockwise postoperative relapse^4,21,22^. CCWR of MMC can cause stretching of the pterygomasseteric sling, which in turn produces tension on the area. These, in combination with the inferior and posterior traction forces originating from the suprahyoid muscles, tends to rotate the MMC clockwise and promote the relapse. To correct these problems, some technical modifications have been proposed, such as the detachment and sectioning of the pterygomasseteric sling^3^, as well as performing inverted L osteotomy. These interventions can preserve the connection of the pterygoid muscles with the proximal mandibular stump. Meanwhile, the use of computer-aided surgical simulation technology could predicting the treatment outcomes in an accurate and effective way, giving reference for surgeons and patients when making a trade-off between the relapse risk and aesthetics outcomes^23–25^.

In this study, twenty-three patients had a series of TMJ health problems. The magnetic resonance imaging confirmed the different degrees of disc displacement and condylar resorption in these patients, which may contribute to the mandibular-body segments relapse. The Position-Posture measuring method established the mandibular coordinate system presuming that the condylion and coracoid process points remain unchanged after surgery and the positional relationship of the ramus bilaterally is stable. In order to minimize the errors, the plane of angle between the right and left ramus was measured to verify its reliability, with the maximum value under 2° and no statistically difference (*P* > *0.05*). This shows the reliability of the coordinate system of the mandibular model.

The 3D CT scans of patients at different time points were chosen for evaluation because of the excellent visualization of craniofacial skeleton. Although the CT scan examination has higher amounts of radiation than 2D cephalometry and cone beam computed tomography, is generally extremely useful and necessary for pre-surgical simulation and postoperative observation for hard and soft tissue evaluation in clinical application^24^. The 3D CT scan gives more information about important microanatomy structures involving in orthognathic surgery and more detail about soft tissue changes, which could be essential to establish a universal 3D cephalometric system for both bony structures and soft tissue in the future. Meanwhile, this study established a novel measurement method to analyze the postsurgical stability of hard tissues according to the experience of the clinicians. To promote its clinical application, we recommend to select more landmarks to serve as references. The clinical significance will be used to describe the position and posture of the craniofacial hard tissue. We suggest that further research is needed to establish the evaluation systems of bone and soft tissue segments by using the Position-Posture measuring method.

In conclusion, this Position-Posture measuring method has the advantage of revealing the linear and angular changes of the maxillary and mandibular models with great accuracy. It can also help in analyzing the three-dimensional postoperative relapse of hard tissues. By using this method, we can predict the skeletal stability of the maxilla after the counter-clockwise rotation procedure. This can also reveal the tendency for a clockwise relapse of the mandible in the skeletal class II patient.

## Methods

The study was conducted in accordance with the World Medical Association Declaration of Helsinki on medical research ethics. All experimental protocols in this clinical retrospective study were approved by the Ethics Committee of Shanghai Ninth People’s Hospital.

### Patients

This study was performed in the Department of Oral and Craniomaxillofacial Surgery, Ninth People’s Hospital, Shanghai Jiao Tong University School of Medicine, Shanghai, China. From January 2015 to October 2016, 25 patients (5 males and 20 females) with skeletal class II deformity have undergone orthognathic surgery with the CCWR procedure.

The following were the inclusion criteria used to select the candidate patients:

1. preoperative diagnosis of skeletal class II deformity;
2. a minimum age of 18 years old;
3. the use of rigid fixation.

Patients were excluded using the following exclusion criteria:

1. patients diagnosed having a syndrome;
2. those with either growth or mental retardation.

All patients included in the study accomplished and signed an informed consent.

Pre and post-operative CT (Light speed 32, GE, UK: 1.25 mm slice thickness) were obtained for all patients 1 week prior to surgery(T0), 3–7days(T1), 3 months(T2), and 6 months(T3) after surgery.

### Surgical procedure

Commercially available surgical simulation software SimPlant Pro 11.4 (Materialise Dental, Belgium) was used for the pre-operative planning. All of the personalized virtual surgical planning included counterclockwise rotation of the MMC. All patients underwent orthognathic surgery of the one-piece Lefort I osteotomy and bilateral sagittal split ramus osteotomy (BSSRO) under the guidance of a 3D printed occlusion splints.

### Measuring method

**Figure 1:**
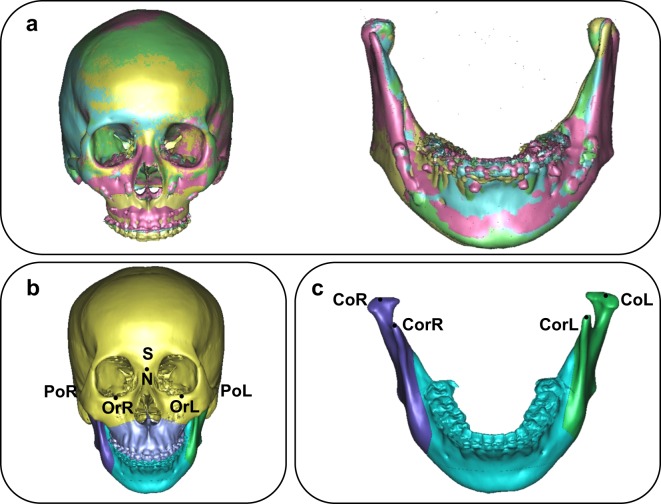
(**a**) Cranio-maxillofacial and mandibular models of T0-T3 with surface-best-fit method (yellow: T0, pink: T1, blue: T2, green: T3). (**b**) Bony landmarks of cranio-maxiollofacial were chosen to establish coordinate systems. (**c**) Bony landmarks of mandibular models were chosen to establish coordinate systems.

The skull model, was separated into two models, the cranio-maxiollofacial model and mandibular model. Through the use of the surface-best fit registration method^20^, the 3D cranio-maxiollofacial models and mandibular models of T1, T2, and T3 were superimposed on T0 ones (Fig. 1a) consecutively with the aid of the SimPlant Pro software.

Bony landmarks of the cranio-maxiollofacial models (Fig. 1b) and mandibular models (Fig. 1c) were chosen by experienced surgeons to establish the coordinate systems separately (Table 2).

**Table 2:** Definition of skeletal landmarks.

Abbreviation	Landmark	Anatomical site
S	Sella	the geometric center of the sella turcica
N	Nasion	most posterior point on curvature between frontal bone and nasal bone in midsagittal plane
Or L/R	Orbitale	lowest point on infraorbital margin of each orbit
Po L/R	Porion	highest midpoint on roof of external auditory meatus
A	Subspinale	most posterior point on curve between ANS and prosthion
SPC	Superior central prosthion	most anterior point in the midline on the alveolar process between upper central incisors
SP6L/SP6R	Superior molar prosthion	most anterior point in the midline on the alveolar process of the upper left/right first molar
B	Supramental	most posterior point of bony curvature of mandible below infradentale and above pogonion
IPC	Inferior central prosthion	most anterior point in the midline on the alveolar process between lower central incisors
IP6L/IP6R	Inferior molar prosthion	most anterior point in the midline on the alveolar process of the lower left/right first molar
Co L/R	condylion	most superior point of left/right ondylar head surface
Cor L/R	coracoid process	most superior point of left/right coracoid process surface
GoL/R	Gonion L/R	most inferior, posterior, and lateral point on the external angle of the mandible

#### Coordinate system of cranio-maxiollofacial model

**Figure 2:**
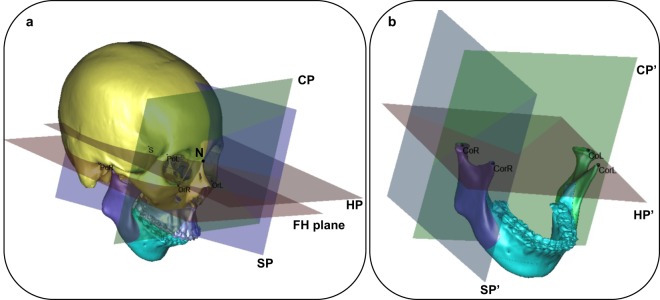
(**a**) Coordinate systems of cranio-maxiollofacial model with 45°angle. (**b**) Coordinate systems of mandibular model with 45°angle.

Cranial segment, which remain unchanged before and after surgery, was defined as the reference coordinate system of the 3D cranio-maxiollofacial model (Fig. 2a).

The horizontal reference plane (HP): parallel to the FH plane, which was constructed on both sides of Po and left side Or, passing through N.

The midsagittal plane (SP): perpendicular to the horizontal plane passing through N and S.

The coronal plane (CP): perpendicular to the horizontal and midsagittal plane passing through N.

N point was defined as the original point of the coordinate system.

#### Coordinate system of mandibular model

For T1, T2 and T3 mandibular models, Co L/R and Cor L/R remain unchanged after surgery, which could be used to defined the reference coordinate system of mandibular model (Fig. 2b).

The mandible-body horizontal reference plane (HP’) was constructed on Co L/R and CorR.

The mandible-body midsagittal plane (SP’) was drawn perpendicular to the horizontal plane passing through CoR and CorR.

The mandible-body coronal plane (CP’) was drawn perpendicular to the horizontal and midsagittal plane passing through CoR.

CoR was defined as the original point of the coordinate system.

In order to precisely measure and analyze the changes, the target cranio-maxiollofacial model was defined as cranial segment and maxillary segment, and the target mandibular model was referred as mandibular-body segment and bilateral ramus segments. We can choose a series of remarkable anatomical landmarks for describing the position of each segments, while three mutually perpendicular planes for describing their posture were also used. Therefore, position and posture of each segment could be defined separately as follows:

Cranial segment: position/posture: Cranial segment, which remain unchanged before and after surgery, was defined as the reference coordinate system of the 3D cranio-maxiollofacial model. So the landmarks of cranio-maxiollofacial model (Fig. 1b) was defined as the position of cranial segment, and the three coordinate planes (Fig. 2a) as its posture.

Maxillary segment: position: In order to describe the position of maxilla, several clinically significant anatomical landmarks were chosen by experienced surgeon. Skeletal landmarks A, SPC, SP6L and SP6R (Table 2) were chosen to describe the position of maxillary segment.

**Figure 3:**
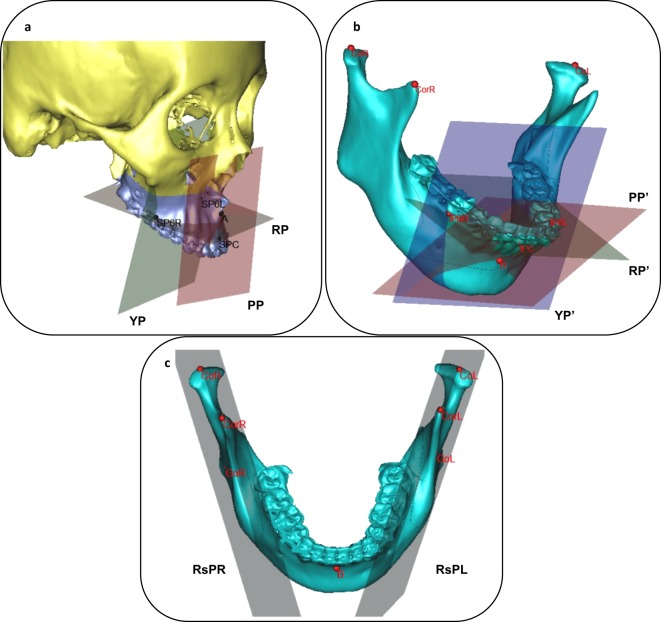
(**a**) Posture plane (PP, RP and YP) of maxillary segments. (**b**) Posture plane(PP, RP and YP) of mandible-body segment. (**a**) Bony landmarks and posture planes of bilateral ramus segments.

Maxillary segment: posture: Based on the these landmarks, we defined 3 posture planes (Fig. 3a).

yaw plane (YP): passing through 2 points (SP6L, SP6R) and normal to HP;

pitch plane (PP): passing through 2 points (A, SPC) and normal to SP

roll plan (RP): passing through 2 points (SP6L, SP6R) and normal to CP

Mandible-body segment: position: In order to describe the position of mandibular-body, several clinically significant anatomical landmarks were chosen by experienced surgeon. Skeletal landmarks B, IPC, IP6L and IP6R (Table 1) were chosen to describe the position of mandibular-body segment.

Mandible-body segment: posture: Based on the these landmarks, we defined 3 posture planes (Fig. 3b).

yaw plane (YP’): passing through 2 points (IP6L, IP6R) and normal to HP’;

pitch plane (PP’): passing through 2 points (B, IPC) and normal to SP’;

roll plan (RP’): passing through 2 points (IP6L, IP6R) and normal to CP’.

Ramus segments: position: Several clinically significant anatomical landmarks were chosen to describe the position of the ramus segments such as CoL/R, CorL/R and GoL/R. (Table 1).

Ramus segment: posture: Because of the shape of ramus segment, we defined its posture as one plane based on these landmarks (Fig. 3c).

Ramus plane (RsP L/R): passing through 3 points (Co, Cor, Go).

The linear differences of anatomical landmarks could represent the positional change of target maxillary segments and mandibular-body segments on each three-dimensional coordinate system (X, Y, Z) (Table 3).

**Table 3:** Coordinate value of maxillary&mandibular landmarks in 3-D coordinate system.

value	A	SPC	SP6L	SP6R	B	IPC	IP6L	IP6R
X	A-SP	SPC-SP	SP6L-SP	SP6R-SP	B-SP’	IPC-SP’	IP6L-SP’	IP6R-SP’
Y	A-HP	SPC-HP	SP6L-HP	SP6R-HP	B-HP’	IPC-HP’	IP6L-HP’	IP6R-HP’
Z	A-CP	SPC-CP	SP6L-CP	SP6R-CP	B-CP’	IPC-CP’	IP6L-CP’	IP6R-CP’

**Figure 4:**
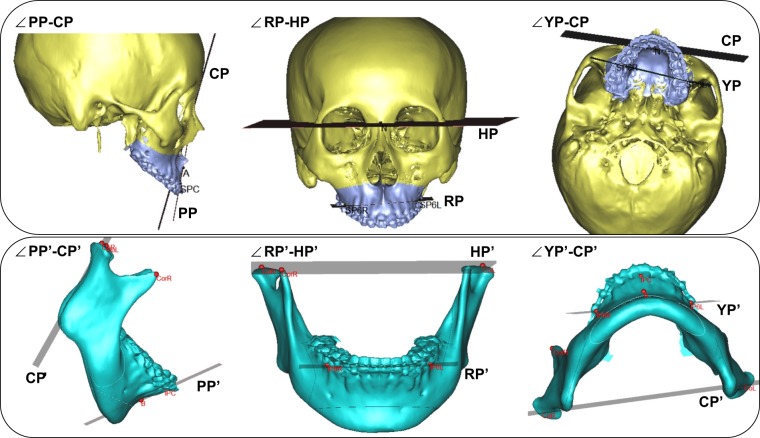
Definition of angle between the posture planes (PP, RP and YP) and reference planes on cranio-maxiollofacial model and mandibular model.

We also measured the angle differences between the posture planes and reference planes of the cranio-maxiollofacial and mandibular model (Table 4, Fig. 4).

**Table 4:** Definition of angle between the posture planes and reference planes.

Angle	Definition
maxillary∠PP-CP (deg)	the angle between the maxillary CP and maxillary pitch plane, which passing through 2 points (A, SPC) and normal to SP
maxillary∠RP-HP (deg)	the angle between the maxillary HP and maxillary roll plane, which passing through 2 points (SP6L, SP6R) and normal to CP
maxillary∠YP-CP (deg)	the angle between the maxillary CP and maxillary yaw plane, which passing through 2 points (SP6L, SP6R) and normal to HP
mandibular∠PP’-CP’ (deg)	the angle between the mandibular CP’ and mandibular pitch plane, which passing through 2 points (B, IPC) and normal to SP’
mandibular∠RP’-HP’ (deg)	the angle between the mandibular HP’ and mandibular roll plane, which passing through 2 points (IP6L, IP6R) and normal to CP’
mandibular∠YP’-CP’ (deg)	the angle between the mandibular CP’ and mandibular yaw plane, which passing through 2 points (IP6L, IP6R) and normal to HP’

### Statistical analysis

All measurements were tabulated and sequenced in the different time frame. Differences between groups were considered significant at p ≤ 0.05. One-way ANOVA was used for statistical analysis. All calculations were carried out using the software package SPSS 19.0 (IBM, American).

## Data Availability

The data that supports the findings reported herein are available upon request from the corresponding author.
